# Management of Pancreatico-duodenal arterio-venous malformation

**DOI:** 10.1186/s42155-021-00269-9

**Published:** 2022-01-03

**Authors:** Clement Marcelin, Auh Whan Park, Patrick Gilbert, Louis Bouchard, Eric Therasse, Pierre Perreault, Marie France Giroux, Gilles Soulez

**Affiliations:** 1 CHUM Université de Montréal, Montreal, Québec Canada; 2 Department of Radiology, UVA Health, Charlottsville, VA, USA

**Keywords:** Embolization, AVM, Pancreas, Percutaneous

## Abstract

**Purpose:**

To describe the interventional management and clinical outcome of pancreatico-duodenal arterio-venous malformations (PDAVMs).

**Material and Methods:**

Seven patients presenting a PDAVM (6 women, 1 male; mean age: 61) were retrospectively reviewed. Technical, clinical success and complications of embolization and surgical management of symptomatic PDAVMs were assessed. Technical success was defined as a complete occlusion of the PDAVM and clinical success as no clinical symptom or recurrence during follow-up. Patients with asymptomatic PDAVMs were followed clinically, by Doppler ultrasound and CT-angiography.

**Results:**

Mean follow-up time was 69 months (15-180). Five symptomatic patients presented with upper gastrointestinal bleeding (*n*=3), ascites (*n*=1), and abdominal pain (*n*=1). Two patients were asymptomatic. The PDAVMs were classified as follow: Yakes I (1), IIIa (2), IIIb (3) and IV (1). Five symptomatic patients were treated with 9 embolization sessions with arterial approach (onyx®, glue, coils) in 7 and venous approach in 2 (plugs, coils, covered stents, STS foam and onyx®). Technical success of embolization was 60% (3/5). Devascularization was incomplete for 2 Yakes IIIB patients. Clinical success of embolization was estimated at 80% (4/5) as one patient required additional surgery (Whipple) because of persistent bleeding. One splenic vein thrombosis was treated successfully by mechanical thrombectomy and heparin. No recurrence occurred during follow-up. No progression was documented in asymptomatic patients.

**Conclusion:**

Embolization of symptomatic PDAVMs is effective and surgery should be performed in second intention. Complete devascularization is more difficult to obtain in Yakes III PDAVM.

## Introduction

Pancreatico-duodenal arterio-venous malformations (PDAVMs) are rare but a proper diagnosis is important to differentiate them from hypervascular tumors (Shearer et al., 2011) . PDAVMs are mostly congenital, and can be associated with Hereditary Hemorrhagic Telangiectasia, commonly known as Osler-Weber-Rendu Syndrome (Lacout et al., 2010).

PDAVMs are often asymptomatic, but complications can arise, such as gastrointestinal bleeding, abdominal pain, portal hypertension and pancreatitis (Koito et al., 2001). CT is useful to make the diagnosis and identify feeding arteries and draining veins (Ogawa et al., 2009), however arteriography remains the gold standard.

There is no consensus on the management of asymptomatic PDAVMs (Chou et al., 2013) . However, symptomatic PDAVMs can be treated by embolization and/or surgery (Chou et al., 2013; Song et al., 2012).

In previous case reports PDAVM embolization was technically successful in 84% of cases and led to clinical improvement in 100% of patients (Cassinotto & Lapuyade, 2015; Yamamoto et al., 2008; Frenk et al., 2016; Grasso et al., 2012) . Surgery is effective (Song et al., 2012) but carries a high risk of intraoperative bleeding, and severe complications (Wang et al., 2018).

Yakes and Cho classification helps for determining proper treatment of patients affected by AVMs, by describing the angioarchitecture of the AVM (Soulez et al., 2019; Yakes & Baumgartner, 2014). It will dictate the approach to eradicate the nidus of the AVM (intra-arterial, venous retrograde or direct puncture). Careful selection of the approach is important to reach the nidus while minimizing the risk of reflux of embolizing agents into normal arteries and veins.

The purpose of study is to present a series of PDAVMs and describe the safety and the efficacy of embolization according to the Yakes classification.

## Material and methods

### Study design

The Institutional Review Board approved this study and waived signed informed consent for this retrospective study. Data were collected through review of medical and imaging reports.

All patients with a diagnosis of PDAVM between 2000 and 2019 were included. A search with the following index terms was performed in the radiological information system: arteriovenous malformation and pancreas or duodenum, embolization, CT, Doppler ultrasound, CT, CT angiography.

#### Transarterial embolization

For all patients having embolization, transarterial procedures were performed under local anesthesia and conscious sedation. After percutaneous introduction of a 5-Fr sheath in the right or left femoral artery, the celiac trunk and the superior mesenteric artery (SMA) were selectively catheterized using a 5F Chuang catheter and a hydrophilic guidewire 0.035 (Terumo**®**, Tokyo, Japan). Arteriography was performed to evaluate different feeders, localize the nidus, and visualize draining vein(s).

**Fig. 1:**
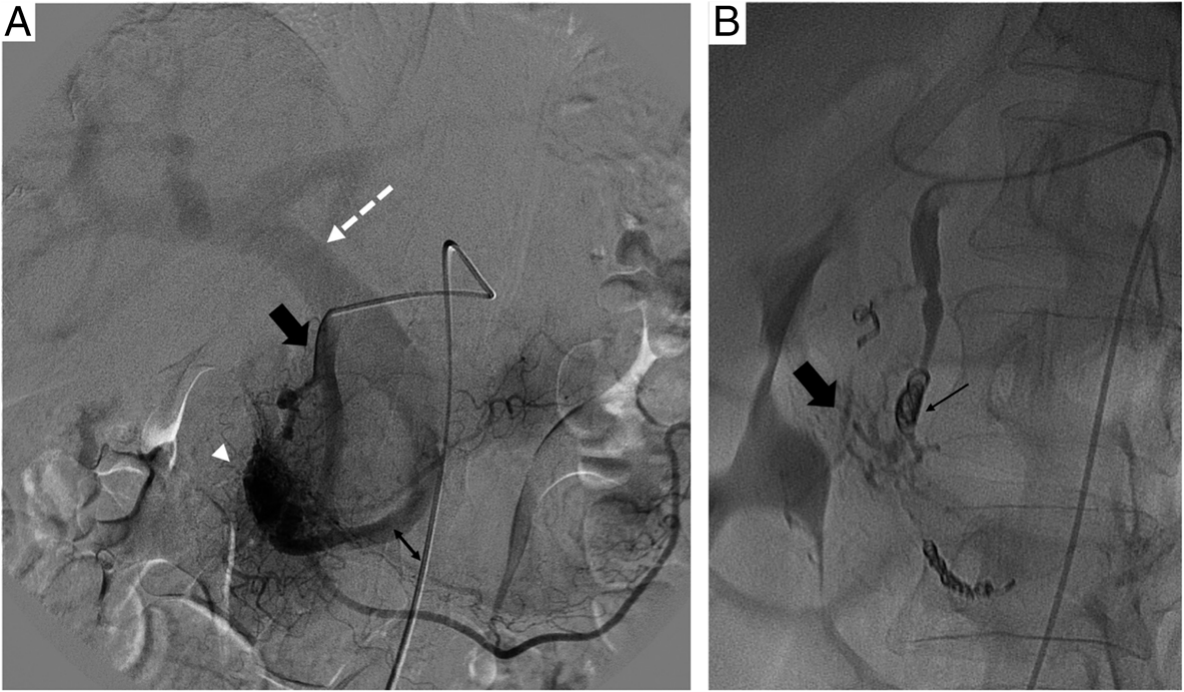
A 79 yo women with upper GI bleeding. A- Arteriography showed a **type IIIa** pancreatic AVM, vascularized by the gastro-duodenal artery (black arrow), connected to the gastro-duodenal vein (double arrow) with a nidus (arrowhead), draining into the portal vein (dotted arrow). B- After embolization of the gastroduodenal artery with coils (black arrow) and the nidus with onyx (large black arrow), opacification of the celiac trunk showed no residual vascularization of the AVM.

The nidus was accessed first by a trans-arterial approach using a microcatheter, either a Progreat Terumo® 2.4 Fr, Tokyo, Japan) or a Renegade (Boston Scientific®, USA), as close as possible to the nidus of the vascular malformation. The arterial feeders were embolized with a liquid agent, chosen based on the operator experience. Ethylene Vinyl Alcohol Copolymer (Onyx 18®, ev3 Irvine, CA, USA) (Fig. 1) or glue (Histoacryl; Braun Germany or Glubran; GEM, Italy) with lipiodol®(Guerbet, France) dilution between 1-2 and 1-3, or 300-500μm PVA particles (when onyx® was not available). Coils (Concerto®, Medtronic, Target® 360 Stryker, Fremont, CA, and Azur® CX 18, Terumo) were used to protect distal arteries not feeding the AVM, to complete proximal embolization following distal liquid embolization or to decrease arterial flow in combination with a transvenous approach.

#### Transvenous embolization

Transvenous procedures were performed in 2 patients under general anesthesia because of failure of previous trans-arterial embolization.

**Fig. 2:**
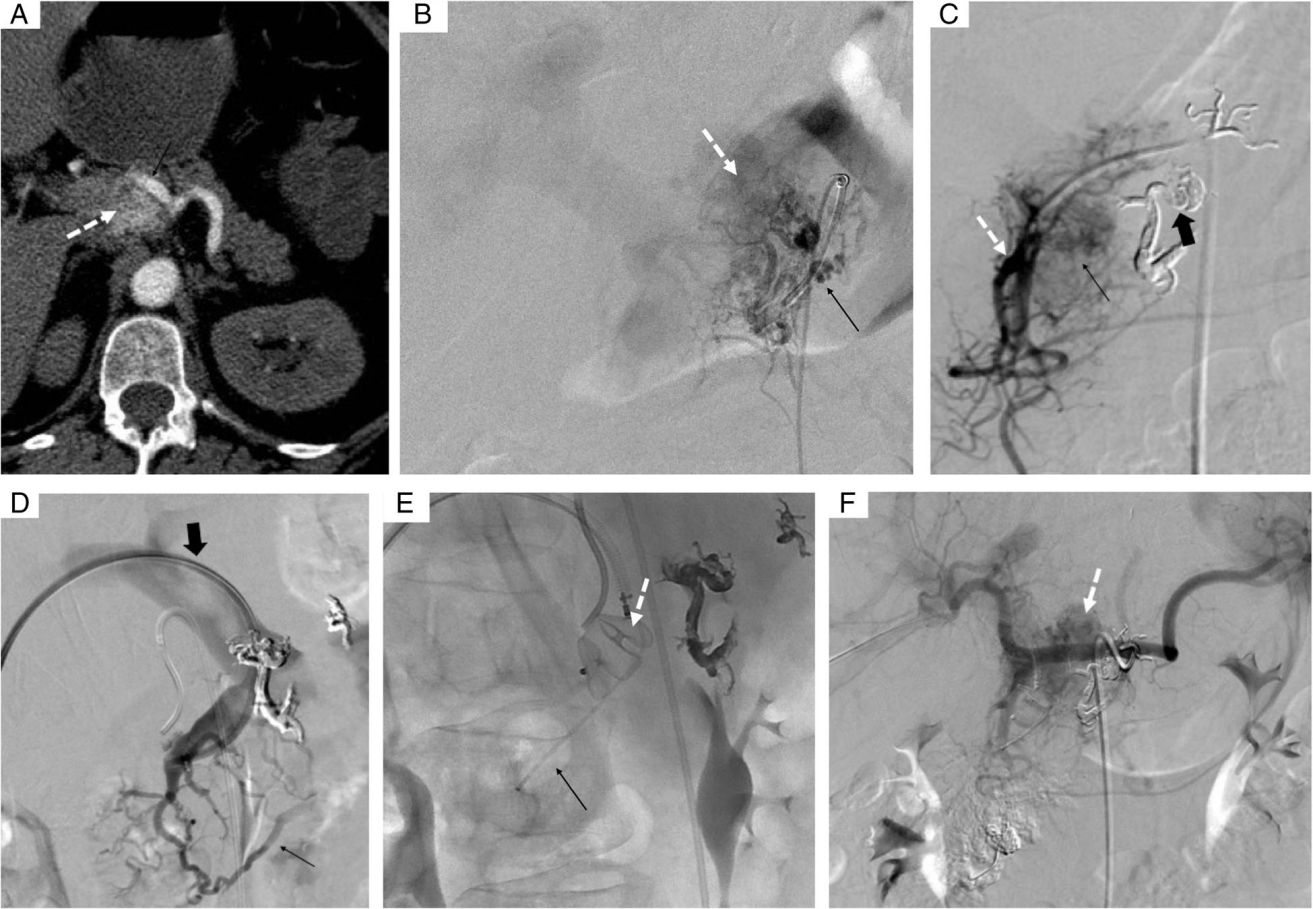
A 57 yo women with upper GI bleeding. A- CT scan showed a nidus in the pancreatic head (dotted arrow). B- Selective angiography of the pancreatic dorsal artery (black arrow) showing a type IIIB pancreatic AVM, with multiple feeding arteries draining in an aneurysmal vein (white arrow) both draining into the portal vein. C- Selective angiography of the gastroduodenal artery showing multiple arterial collaterals (black arrow), vascularized by multiples branches of the postero-superior and antero-superior pancreatico-duodenal arteries (white arrow). Embolization with Onyx® of the dorsal pancreatic artery (large black arrow). D- Portal venous access showed an enlarged pancreatic vein (black arrow) draining into the portal vein (big black arrow). E- Pressure cooker technique: proximal embolization of the draining gastroduodenal vein using a plug (white arrow), and then distal embolization with STS using a microcatheter distal to the Plug in order to reflux into the nidus (black arrow) of the AVM. F- Selective angiography of the celiac trunk showing a residual pancreatic AVM (arrow).

**Fig. 3:**
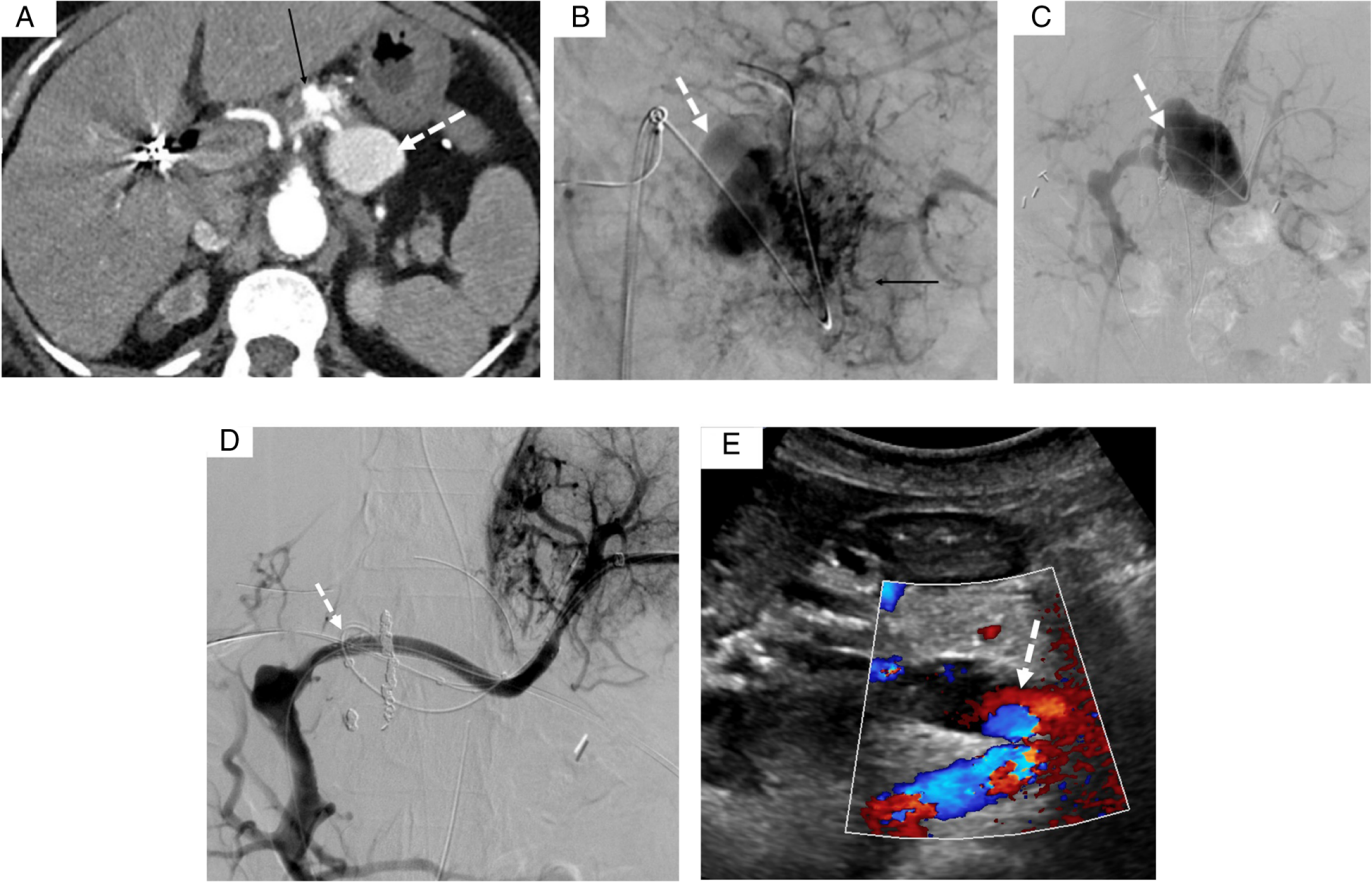
A 66 yo women with a cryptogenic cirrhosis who underwent previous abdominal surgery for colorectal carcinoma and chronic portal vein thrombosis presented recurrent ascites and chronic pancreatitis. A CT scans showing a Yakes type IIIa pancreatic AVM (arrow), with an aneurysmal splenic vein (dashed arrow). B Selective angiography of the splenic artery showed a pancreatic AVM, vascularized by the dorsal pancreatic artery (arrow), splenic artery, left gastric artery, and connected to an aneurysmal splenic vein (dashed arrow). C After puncture of splenic vein, venography showing the aneurysmal splenic vein draining into the gastroduodenal and mesenteric veins because of the preexisting portal thrombosis (dashed arrow). D Insertion of a covered stent in the splenic vein (dashed arrow) by a transplenic access and embolization using Onyx® and coils after direct puncture of the aneurysm. The patient had subsequent splenic venous thrombosis which was successfully treated by mechanical thrombectomy and heparin infusion. E Doppler ultrasound at 1 year showed permeability of the splenic and portal veins (dashed arrow), with no residual AVM.

In one case, the AVM located in the head of the pancreas was draining in the gastroduodenal vein. A percutaneous portal access was performed under ultrasound with a micropuncture set. A coaxial technique was used with deployment of an Amplatzer® plug (Amplatzer®, St Jude medical, Plymouth, Minnesotta) in the proximal portion of the gastroduodenal vein. Using a microcatheter (Progreat® 2.7, Terumo, Tokyo, Japan) advanced distally to the plug, the connection between the gastroduodenal vein and the superior mesenteric vein was coiled to prevent reflux in the portal system. Then, the vein was embolized with foam made of two ml of Sodium Tetradecyl Sulfate (STS) 3% (60mg) mixed with 1.5 ml lipiodol and 4ml room air injected distally to the Amplatzer® plug using the pressure cooker technique to reflux into the nidus (Chapot et al., 2014) (Fig. 2). In the second case, the AVM involving the tail of the pancreas was draining into the splenic vein, which was aneurysmal (Fig. 3). Since this patient has a chronic portal thrombosis, a transplenic access was performed. Two covered stent grafts were deployed (Viabahn, Gore, Newark, Deleware) of 6 mm X 7.5 cm in the splenic vein and of 8 mm X 2cm at the spleno-mesenteric vein junction) to exclude the venous aneurysm while maintaining its patency. The aneurysm sac was then embolized using Onyx® and coils after direct puncture of the aneurysm.

#### Image analysis

**Fig. 4:**
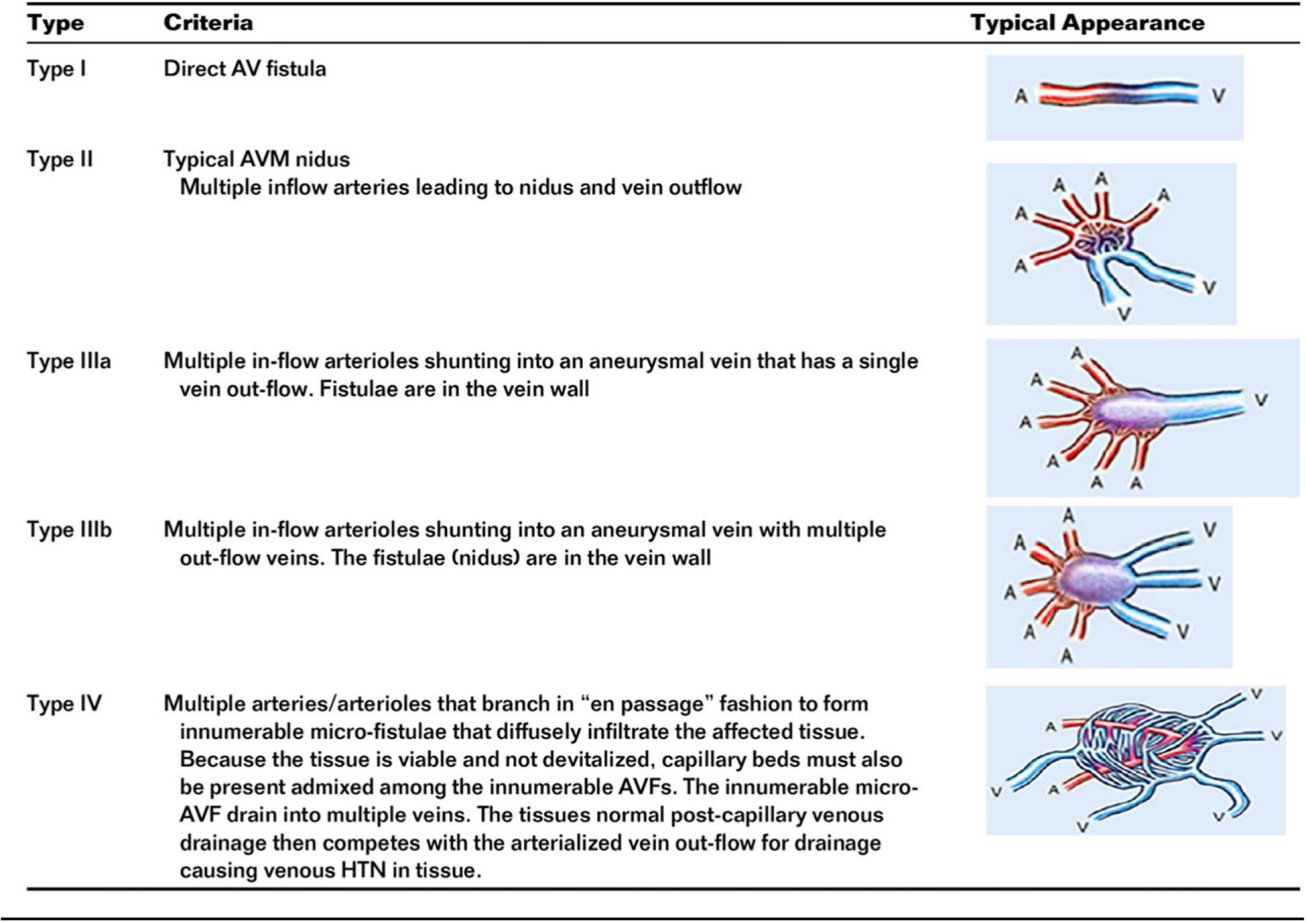
Yakes AVM classification (Soulez et al., 2019)

The PDAVMs were classified according to the Yakes classification (Yakes & Baumgartner, 2014) (Fig. 4).

Technical success was defined as a complete occlusion of the PDAVM on the CT imaging on the follow-up. Clinical success was defined as resolution of symptoms after the embolization during the follow-up.

### Follow-up

Clinical and imaging follow up was performed for all patients at the interventional radiology clinic. All CTs had at least an arterial and portal phase. For patients with an active bleeding a delayed venous phase was also performed. On CT, the criteria for diagnosing the AVM were the presence of enlarged arterial feeders and draining vein and the early filling of the portal system on the arterial phase.

A routine post-embolization contrast-enhanced CT scan with non-contrast, arterial and portal phases, and doppler ultrasound were performed 1 to 6 months after the embolization. Additional intervention was performed in case of PDAVM recurrence. Asymptomatic patients were followed for a mean period of 2 years and had a control CT scan and doppler ultrasound at last follow-up. The utility of Doppler ultrasound is to document the magnitude of AV fistulae (aliasing in the shunting area and arterialization of the portal flow).

## Results

### Patients

Patient characteristics are summarized in Table 1. From 2000 to 2019, seven consecutive patients presenting a PDAVM (6 women, 1 man; mean age: 61.1, range 43-79) were analyzed. No patients were excluded during the study period. Two asymptomatic patients had no intervention and close imaging and clinical follow-up. Five symptomatic patients (2 massive upper gastrointestinal bleeding in emergency and 1 with repeated upper gastrointestinal bleeding for 3 patients, ascites for one, and persistent and resistant abdominal pain for one were treated with first intention embolization (1 male and 4 female). Patients presenting with massive upper gastrointestinal bleeding were initially imaged with CT (2 emergency procedure), and the others were imaged with CT and Doppler ultrasound. Mean follow-up time was 69 months (15-180).

**Table 1:** Patients characteristics

Patient characteristics	Mean (range) or N (%)
Average age in years (range)	61.1 (range 43-79)
Gender	
-Male	1
-Female	6
History of abdominal surgery, portal vein thrombosis, cirrhosis or pancreatitis	1
Symptoms	
-hemorrhage	5
-ascites	1
-abdominal pain	1
-none	2
Classification Yakes	
-I	1
-IIa	-
-IIb	-
-IIIa	2
-IIIb	3
-IV	1
Localization	
-head	3
-isthmus + head	2
-head+isthmus+body	1
-body + isthmus	1
Mean size of the nidus (mm)	24.5 (range 20-30)
Treatment	
-none	2
-embolization	5
-surgery	1

### Imaging findings

On CT and catheter angiography, the feeding arteries included the gastroduodenal artery (*n*=1), the anterior superior pancreatico-duodenal artery (*n*=1), the posterior superior pancreatico-duodenal arteries (*n*=4), the anterior inferior pancreatico-duodenal artery (*n*=1), the posterior inferior pancreatico-duodenal arteries (*n*=5), the great pancreatic arteries (pancreatica magna) (*n*=2), the dorsal pancreatic artery, the splenic artery (*n*=1), the hepatic artery (*n*=1), the left gastric artery (*n*=1).

Draining veins included the gastroduodenal vein (*n*=5), the splenic vein (*n*=2), and the right gastric vein (*n*=1).

### Clinical outcome

Technical success of embolization was estimated at 60% (3/5). Devascularization was incomplete for 2 patients (25% for one patient, and 75% for one patient), both in Yakes IIIB PDAVMs. Clinical success of embolization was estimated at 80% (4/5) (Yakes 1, 2 Yakes 3a, Yakes 3b) as one patient (Yakes IIIB) required additional surgery (Whipple) because of persistent bleeding. This patient had a partial embolization (successive arterial and venous approaches) estimated at 25% devascularization.

One splenic vein thrombosis (Yakes IIIA), occurred after a stenting of aneurysmal splenic vein draining the AVM. It was successfully treated by mechanical thrombectomy followed by an overnight local infusion of heparin. The splenic vein thrombosis was treated to prevent an additional segmental portal hypertension to a chronic portal thrombosis with inherent risk of gastric or gastroesophageal bleeding.

No recurrence occurred during clinical and CT imaging follow-ups. One patient (Yakes IIIB) had 25% residual AVM without symptom recurrence during the follow-up.

The patient with splenic vein thrombosis and preexisting portal thrombosis was anticoagulated for 12 months with good patency of the covered stent-grafts, without recurrence of AVM. There was no other complication and no evidence of deterioration of liver, pancreatic and renal function on blood tests. Patient hospitalization ranged between 24 hours and 7 days.

Asymptomatic patients presented with a Yakes IIIb and IV, and had no evidence of AVM progression and did not require additional treatment.

## Discussion

In this study complete regression of symptoms of the AVM was reported at 80% while technical success of embolization was 60%. One of the 2 Yakes IIIB patients with incomplete embolization (25%) required additional surgery whereas the other with a 75% devascularization had a good evolution with conservative treatment.

Classification of AVMs is useful for planning optimal therapeutic approach, and predicting therapeutic outcomes (Yakes et al., 1996; Cho et al., 2006). Cho et al. (2006) demonstrated better outcome in type II Cho AVMs involving extremities or the thoraco-abdominal region.

Yakes classification was preferred to Cho classification in this study because it was more representative of the angioarchitecture observed on imaging. The Yakes classification is more comprehensive especially the distinction between type IIIa and III b which applied for 42% (3/7) of our patients.

Six of seven patients had no history of previous abdominal surgery, splenic or portal vein thrombosis or pancreatitis, these patients probably had in-born AVMs discovered at the adult age. The patient showed in Fig. 3 who had a previous abdominal surgery, portal thrombosis and chronic pancreatitis could have an acquired AVM.

Ideally, the embolization endpoint is to obtain a complete nidus occlusion of the PDAVM. Transarterial approach was used in first intention to embolize the PDAVM. Transvenous approach was selected in second intention following failure of transarterial approaches or when multiple arterial feeders were present with a dominant draining vein.

Transvenous approach was useful, however embolization should be performed carefully to minimize the risk of splenic or portal thrombosis. We observed one case of the latter, which was successfully managed by mechanical thrombectomy and local heparin infusion.

Different embolic agents have been used in previous reports: particles and ethanol were described with good technical success (Frenk et al., 2016), embolization with Onyx® showed complete regression of PDAVMs (Cassinotto & Lapuyade, 2015; Grasso et al., 2012), and Glue utilized as well (Tatsuta et al., 2014). Liquid embolic agents are the most useful. Onyx® provides the best control for liquid agent and should be preferred to minimize the risk of non-target embolization (Guimaraes & Wooster, 2011). The speed of polymerization of glue is more difficult to predict and the slow injection rate of Onyx® give more chance to the operator to prevent non target embolization (Li & Barthes-Biesel, 2017). Glue had a higher risk of bowel infarction and glue reflux into other vessels may result in non-target embolization. On the venous side, the combination of mechanical occlusion with coils or plugs with a liquid agent is recommended (Soulez et al., 2019; Lee et al., 2019). Insertion of a covered stent-graft combined with embolization of a venous aneurysm is an elegant approach to maintain the patency of splanchnic veins in case of an aneurysmal draining vein ( Yakes IIB, IIIA) (Beyer et al., 2015). Ethanol embolization has the potential to cure the AVM, however it carries a higher-risk of non-target embolization and potential gastroduodenal ulcer or pancreatitis.

Reported complications included gastric ulcer, pancreatitis, portal vein thrombosis, bowel ischemia (Lee, 2020). The occurrence of duodenal ulceration following the arterial approach being the most frequent (Cassinotto & Lapuyade, 2015; Grasso et al., 2012). Selective TAE with NBCA in the pancreas caused localized ischemic necrosis without clinically significant pancreatitis in a swine model (Okada et al., 2012).

The transvenous approach for PDAVM was not reported yet. This approach carries a risk of portal, mesenteric or splenic vein thrombosis. It is important to control reflux of liquid embolic agent into the portal system by occluding potential re-entry in the portal system. This could be achieved by occluding draining veins connected to the portal system with plugs or coils. Intra-nidal sclerotherapy by direct puncture was not performed because the AVM were deeply located and difficult to image under ultrasound or non-contrast or delayed contrast enhanced CT. Moreover, this requires to puncture through digestive structures in most cases.

In an emergent setting, microparticles were used because the operator was not used to liquid embolics. Using 300-500um particles, there was no significant complication as previously reported by Aina et al for gastroduodenal bleeding (Aina et al., 2001).

Additional surgery could be proposed in case of persistent of symptomatic AVM after embolization.

Radiotherapy has been reported (Kishi et al., 2011), in asymptomatic patients, showing shrinkage of the AVM. TIPS was described in case of failure of PDAVM embolization, with good efficacy (Hayashi et al., 1998).

Total or partial pancreatectomy is the only complete cure treatment, however there is a risk of massive intraoperative bleeding, hypoglycemia, and pancreatic juice leakage (Song et al., 2012).

The limitations of the study were the small number of patients and the retrospective design using different embolic agents. Particles were used only in emergency when the interventionalist in charge was not comfortable with the use of liquid embolics. Imaging follow-up were made by CT, thus, small residual AVMs with minimal AV shunting may have been overlooked. However, all patients did not have bleeding recurrence or evidence of portal hypertension during the follow-up. PDAVMs are rare, making a prospective study impossible in a reasonable time frame.

In conclusion, embolization of PDAVMs is a safe approach and can be effective. In case of persistent symptomatic PDAVM after embolization, surgery is a valid option. Yakes Grade IIIb PDAVM are more challenging and partial devascularization can be observed.

## References

[CR1] Aina R, Oliva VL, Therasse É, Perreault P, Bui BT, Dufresne M-P (2001). Arterial Embolotherapy for Upper Gastrointestinal Hemorrhage: Outcome Assessment. J Vasc Interv Radiol.

[CR2] Beyer LP, Wohlgemuth WA, Uller W, Pregler B, Goessmann H, Niessen C (2015). Percutaneous treatment of symptomatic superior mesenteric vein stenosis using self-expanding nitinol stents. Eur J Radiol.

[CR3] Cassinotto C, Lapuyade B (2015). Pancreatic Arteriovenous Malformation Embolization with Onyx. J Vasc Interv Radiol.

[CR4] Chapot R, Stracke P, Velasco A, Nordmeyer H, Heddier M, Stauder M (2014). The Pressure Cooker Technique for the treatment of brain AVMs. J Neuroradiol.

[CR5] Cho SK, Do YS, Shin SW, Kim D-I, Kim YW, Park KB (2006). Arteriovenous Malformations of the Body and Extremities: Analysis of Therapeutic Outcomes and Approaches According to a Modified Angiographic Classification. J Endovasc Ther.

[CR6] Chou S-C, Shyr Y-M, Wang S-E (2013). Pancreatic Arteriovenous Malformation. J Gastrointest Surg.

[CR7] Frenk NE, Chao TE, Cui J, Fagenholz PJ, Irani Z (2016). Staged Particle and Ethanol Embolotherapy of a Symptomatic Pancreatic Arteriovenous Malformation. J Vasc Interv Radiol.

[CR8] Grasso RF, Cazzato RL, Luppi G, Faiella E, Del Vescovo R, Giurazza F (2012). Pancreatic Arteriovenous Malformation Involving the Duodenum Embolized with Ethylene-Vinyl Alcohol Copolymer (Onyx). Cardiovasc Intervent Radiol.

[CR9] Guimaraes M, Wooster M (2011). Onyx (Ethylene-vinyl Alcohol Copolymer) in Peripheral Applications. Semin Interv Radiol.

[CR10] Hayashi N, Sakai T, Kitagawa M, Inagaki R, Yamamoto T, Ishii Y (1998). Intractable gastrointestinal bleeding caused by pancreatic arteriovenous malformation: successful treatment with transjugular intrahepatic portosystemic shunt. Eur J Radiol.

[CR11] Kishi K, Shirai S, Sato M, Sonomura T (2011). Role of external beam radiotherapy for arteriovenous malformation of the pancreas. Jpn J Radiol.

[CR12] Koito K, Namieno T, Nagakawa T, Ichimura T, Hirokawa N, Mukaiya M (2001). Congenital Arteriovenous Malformation of the Pancreas: Its Diagnostic Features on Images. Pancreas.

[CR13] Lacout A, Pelage J-P, Lesur G, Chinet T, Beauchet A, Roume J (2010). Pancreatic Involvement in Hereditary Hemorrhagic Telangiectasia: Assessment with Multidetector Helical CT. Radiology.

[CR14] Lee SM (2020). Transcatheter Arterial Embolization for Gastrointestinal Bleeding related to Pancreatic Adenocarcinoma: Clinical Efficacy and Predictors of Clinical Outcome. Eur J Radiol.

[CR15] Lee SY, Do YS, Kim CW, Park KB, Kim YH, Cho YJ (2019). Efficacy and Safety of Transvenous Embolization of Type II Renal Arteriovenous Malformations with Coils. J Vasc Interv Radiol.

[CR16] Li YJ, Barthes-Biesel D (2017). Polymerization kinetics of n-butyl cyanoacrylate glues used for vascular embolization. J Mech Behav Biomed Mater.

[CR17] Ogawa H, Itoh S, Mori Y, Suzuki K, Ota T, Naganawa S (2009). Arteriovenous malformation of the pancreas: assessment of clinical and multislice CT features. Abdom Imaging.

[CR18] Okada T, Yamaguchi M, Takahashi T, Izaki K, Uotani K, Sakamoto N (2012). Is Embolization of the Pancreas Safe? Pancreatic Histological Changes after Selective Transcatheter Arterial Embolization with N-Butyl Cyanoacrylate in a Swine Model. Cardiovasc Intervent Radiol.

[CR19] Shearer DD, Demos TC, Sichlau MJ. Pancreatic Arteriovenous Malformation: a case report and literature review. J Radiol Case Rep. 2011;5(8):8–13. 10.3941/jrcr.v5i8.742 PMC3303456 22470807

[CR20] Song KB, Kim SC, Park JB, Kim YH, Jung YS, Kim M-H (2012). Surgical Outcomes of Pancreatic Arteriovenous Malformation in a Single Center and Review of Literature. Pancreas.

[CR21] Soulez G, Gilbert P, Giroux M-F, Racicot J-N, Dubois J (2019). Interventional Management of Arteriovenous Malformations. Tech Vasc Interv Radiol.

[CR22] Tatsuta T, Endo T, Watanabe K, Hasui K, Sawada N, Igarashi G (2014). A Successful Case of Transcatheter Arterial Embolization with N-butyl-2-cyanoacrylate for Pancreatic Arteriovenous Malformation. Intern Med.

[CR23] Wang J, Ma R, Churilov L, Eleftheriou P, Nikfarjam M, Christophi C (2018). The cost of perioperative complications following pancreaticoduodenectomy: A systematic review. Pancreatol Off J Int Assoc Pancreatol IAP Al.

[CR24] Yakes W, Baumgartner I (2014). Interventional treatment of arterio-venous malformations. Gefässchirurgie.

[CR25] Yakes WF, Rossi P, Odink H (1996). How I do it. Arteriovenous malformation management. Cardiovasc Intervent Radiol.

[CR26] Yamamoto K, Tominaga K, Kanke K, Iijima M, Abe A, Shimoda M (2008). Hepatobiliary and pancreatic: Arteriovenous malformation of the pancreas. J Gastroenterol Hepatol.

